# Characterization of *Cronobacter* spp. isolated from food products in Poland and comparative genomic analysis of *Cronobacter sakazakii* isolate MK_10 and a clinical strain associated with a fatal neonatal infection

**DOI:** 10.3389/fmicb.2025.1713963

**Published:** 2025-12-19

**Authors:** Dorota Korsak, Magdalena Szuplewska, Paulina Kozłowska, Kamil Krakowski, Cora Chmielowska, Paweł Wawrzyniak, Elżbieta Maćkiw, Dariusz Bartosik

**Affiliations:** 1 Department of Molecular Microbiology, Institute of Microbiology, Faculty of Biology, University of Warsaw, Warsaw, Poland; 2 Department of Bacterial Genetics, Institute of Microbiology, Faculty of Biology, University of Warsaw, Warsaw, Poland; 3 Laboratory of Structural Bioinformatics, Institute of Evolutionary Biology, Faculty of Biology, University of Warsaw, Warsaw, Poland; 4 Department of Food Safety, National Institute of Public Health NIH, National Research Institute, Warsaw, Poland

**Keywords:** *Cronobacter* spp., *Cronobacter sakazakii*, food contamination, genome structure, virulence plasmids, mobile genetic elements

## Abstract

*Cronobacter* spp. are ubiquitous environmental bacteria associated with infections affecting all age groups, with infants and elderly individuals particularly at risk. We investigated the prevalence of *Cronobacter* spp. in 251 food samples collected in Poland, including raw milk, powdered milk, infant formula, dried herbs and spices, dried teas, and raw vegetables. *Cronobacter* strains were isolated from 26 samples (10.4%) and classified into five species: *C. sakazakii*, *C. muytjensii*, *C. turicensis*, *C. malonaticus* and *C. dublinensis*. The isolates were grouped based on sequence variation of the *fusA* and *rpoB* genes, and further differentiated according to their biochemical profiles. The complete nucleotide sequence of *C. sakazakii* MK_10, a plasmid-rich strain isolated from dried oregano and representing the most prevalent species in the collected strain pool, was obtained. Comparative genomic analysis of MK_10, the first fully sequenced foodborne *C. sakazakii* strain in Poland, with a clinical strain from a fatal neonatal case revealed the presence of a conserved set of genes associated with pathogenic properties. MK_10 carries three extrachromosomal replicons, including two IncFIB plasmids – pCS-MK10_P1 (114 kb), representing the pESA3 family of virulence plasmids, and pCS-MK10_P2 (45.9 kb) carrying a copper homeostasis and silver resistance island (CHASRI). Comparative analyses revealed that the structure of the MK_10 chromosome has been shaped by multiple insertion and deletion events, associated mainly with prophages (e.g., a novel intact prophage related to *Cronobacter* phage ENT47670) and other integrative elements. The MK_10 genome contains only two transposable elements: an isoform of insertion sequence IS*Ehe2* (IS*3* family) and a non-autonomous Tn*3*-family transposon of unique structure – both located within two functional integrative elements (IE), designated IE*Csak_MK10_1* (32.3 kb) and IE*Csak_MK10_2* (32.4 kb). The identified mobile genetic elements, plasmids, and IEs, harbor genes of adaptive value, which potentially contribute to the strain’s environmental fitness and pathogenic potential.

## 1 Introduction

*Cronobacter* spp. (previously known as *Enterobacter sakazakii*) are Gram-negative, rod-shaped, facultative anaerobic bacteria that belong to the family *Enterobacteriaceae* (class *Gammaproteobacteria*). The genus *Cronobacter* currently comprises seven species: *C. sakazakii, C. malonaticus*, *C. turicensis*, *C. universalis*, *C. muytjensii*, *C. dublinensis* and *C. condimenti* (Joseph et al., 2012; Forsythe, 2018). *Cronobacter* spp. are often associated with plant material, which is assumed to be a major source of contamination of food products with these bacteria (Iversen and Forsythe, 2003; Healy et al., 2010). *Cronobacter* strains have been isolated from a broad range of food products, including powdered infant formulas, follow-up formulas, weaning foods, milk, cereals, spices, teas, pasta, breads, cheese, sausage, tofu, kefir, and chocolate, highlighting the risk of foodborne transmission, particularly to infants. Contamination has also been reported in PIF processing plants, underscoring the importance of maintaining strict hygiene during manufacturing. Moreover, these bacteria have been detected in the human nasopharynx and gastrointestinal tract, suggesting the possibility of asymptomatic carriage. Their presence in domestic environments, such as household dust and kitchen utensils, indicates notable environmental persistence and a risk of cross-contamination, while their isolation from hospital settings emphasizes the threat posed to neonates and immunocompromised patients (Baumgartner et al., 2009; Osaili and Forsythe, 2009; Holý and Forsythe, 2014; Fei et al., 2015; Vojkovska et al., 2016; Brandão et al., 2017; Heperkan et al., 2017; Ueda, 2017; Huvarova et al., 2018; Kadlicekova et al., 2018; Ling et al., 2018; McMullan et al., 2018; Ogihara et al., 2019; Pei et al., 2019; Silva et al., 2019; Gan et al., 2021; Cechin et al., 2023; Wagner et al., 2025). It is also postulated that some animals, e.g., rats, flies and cockroaches, are natural vectors enabling the spread of *Cronobacter* spp. between different environments (Holý and Forsythe, 2014).

*Cronobacter* spp. have been recognized as causative agents of many diseases that affect people across a wide age range. *Cronobacter sakazakii* and *C. malonaticus* are the species most frequently isolated from human infections, followed by *C. turicensis* and *C. universalis*, whereas *C. dublinensis*, *C. muytjensii* and *C. condimenti* have little or no clinical relevance (Forsythe, 2018; Kadlicekova et al., 2018). Several case reports describe infections in elderly patients who were previously ill or immunocompromised (Patrick et al., 2014). The spectrum of clinical manifestations associated with these infections is broad, encompassing sepsis, conjunctivitis, aspiration pneumonia (especially in stroke patients), osteomyelitis, diarrhoea, acute cholecystitis, wound infections, catheter-related abscesses, and urinary tract infections (Holý and Forsythe, 2014; Jaradat et al., 2014). However, the majority of cases of *Cronobacter* spp. infection have been reported in infants, especially those < 28 days old who were born pre-term or with a low birth weight (< 2,500 g) (Lai, 2001; Alsonosi et al., 2015; Mousavi et al., 2023). The symptoms of infection include bacteremia or sepsis, meningitis, abscesses, digestive problems, enterocolitis, necrotizing conjunctivitis and tonsillitis. The neurological sequelae may be permanent, and the mortality rate in this group can reach 40–80% (Holý and Forsythe, 2014). Analyses conducted by Strysko et al. (2020) based on data recorded in 24 countries from 1961 to 2018 showed that among 183 invasive *Cronobacter* infections in infants, the majority involved neonates (67%) and 38% died. Because reporting is not mandatory in most countries (and in most of the United States), the true incidence of invasive infant *Cronobacter* infections is unknown. The reason for the high fatality rate caused by *Cronobacter* spp. is not well understood; however, several virulence factors associated with pathogenesis have been identified (Singh et al., 2015; Demirci et al., 2018; Cechin et al., 2023; Mousavi et al., 2023). A common feature among *Cronobacter* spp. is the presence of so-called virulence plasmids, encoding factors that promote infection and disease, including a siderophore mediated iron-uptake system (Grim et al., 2012).

In 2002, the International Commission on Microbiological Specification for Foods (ICMSF) ranked *E. sakazakii* as a “severe hazard for restricted populations, life threatening or substantial chronic sequelae or long duration.” In 2004, the Scientific Panel on Biological Hazards (BIOHAZ Panel) of the European Food Safety Authority (EFSA, 2004) concluded that *E. sakazakii* and *Salmonella* pose the greatest risk in infant formulae, formulae for special medical purposes and follow-on formulae. Current European Union legislation (Commission Regulation No. 2073/2005, Annex I, point 1.24) lays down food safety criteria for *Cronobacter* spp. in dried infant formulae and dried dietary foods for special medical purposes intended for infants below 6 months of age: their absence from 10 g samples during the shelf-life of products placed on the market. The regulation also requires the manufacturers of such products to monitor processing areas and equipment for *Enterobacteriaceae*. Therefore, the national control and monitoring program carried out in Poland includes *Cronobacter* spp. detection in this type of food.

Monitoring of the occurrence of *Cronobacter* spp. is conducted according to the standard EN ISO 22964, which defines a horizontal method for the detection of these bacteria in food products and ingredients intended for human consumption and the feeding of animals, and environmental samples from locations of food production and food handling. Determination of *Cronobacter* species can be achieved using several approaches. Comparative analysis of 16S rRNA genes amplified by PCR allows the identification of *Cronobacter* spp. strains, although it is not always sufficient for species-level discrimination. More precise differentiation can be achieved using MLST (Multilocus Sequence Typing), which is based on several housekeeping genes (*atpD*, *fusA*, *glnS*, *gltB*, *gyrB*, *infB*, and *ppsA*; Baldwin et al., 2009; Joseph et al., 2012), or through whole-genome sequencing (WGS).

The use of antibiotic therapy is generally effective in infections caused by *Cronobacter* spp. (Depardieu et al., 2007), with traditional treatment involving ampicillin in combination with gentamicin or chloramphenicol (Lai, 2001; Lepuschitz et al., 2019). However, due to confirmed resistance of *Cronobacter* isolates to ampicillin and first- and second-generation cephalosporins, current recommendations suggest the use of third-generation cephalosporins or, as a second-line option, carbapenems, in combination with aminoglycosides or trimethoprim/sulfamethoxazole (Lepuschitz et al., 2019). Antimicrobial susceptibility testing is strongly recommended, as numerous multidrug-resistant strains have been reported (CDC (U.S. Centers for Diseases Control and Prevention), 2025).

The initial aim of this study was to determine the occurrence of *Cronobacter* spp. in various foods sampled in Poland. Isolates were identified using biochemical and molecular methods, and characterized in terms of their antibiotic susceptibility, secretion of extracellular polysaccharides and occurrence of the virulence plasmids typical for these bacteria. In addition, to gain a deeper insight into the genetic background of foodborne *Cronobacter* strains, the complete genomic sequence of one *C. sakazakii* strain (MK_10), isolated from oregano leaves, was obtained. Comparative genomic analysis of MK_10 – the first fully sequenced foodborne isolate from Poland – and a clinical strain associated with a fatal neonatal infection enabled the assessment of the pathogenic potential of MK_10 and identified novel mobile genetic elements that may contribute to the emergence of more virulent *C. sakazakii* lineages.

## 2 Materials and methods

### 2.1 Food sample collection

A total of 251 food samples collected in 2020 were tested for the presence of *Cronobacter* spp. Sixty-three raw milk samples and five raw vegetables samples were obtained from local producers and purchased at local marketplaces to reflect products commonly available to consumers. These samples were transported to the laboratory under refrigerated conditions and analyzed immediately upon receipt. Samples of powdered milk (*n* = 10), infant formula (*n* = 45), dried herbs and spices (*n* = 110), and dried teas (*n* = 18) were randomly purchased from various retail outlets to ensure diversity and representativeness of consumer products. These samples, in their original undamaged packaging, were transported to the laboratory and analyzed as soon as possible.

### 2.2 Bacterial strains and culture conditions

The following *Cronobacter* isolates and reference strains were used as controls in assays: *C. sakazakii* ATCC 29544, *C. muytjensii* ATCC 51329, *C. malonaticus* LMG 23826, *C. universalis* NCTC 9529, *C. turicensis* LMG 23827, *C. dublinensis* LMG 23823 and *Escherichia coli* ATCC 25922. Samples of stock cultures in brain heart infusion broth (BHI; Oxoid, Basingstoke, Hampshire, England) containing 20% sterile glycerol were stored at −80 °C. Inocula for experiments were prepared by streaking one loopful of cell suspension from the stock cultures onto plates of solid BHI medium and incubating at 37 ± 1 °C for 24 ± 2 h. The culture plates were then stored at 4 °C for daily use.

### 2.3 Isolation and biochemical characterization of *Cronobacter* spp

The isolation and analysis of presumptive *Cronobacter* strains was performed in accordance with the standard test method described in PN-EN ISO 22964:2017–06. Typical colonies of presumptive *Cronobacter* spp. were picked from Chromogenic Cronobacter Isolation (CCI) agar medium (Biomaxima S. A., Poland), streaked onto Trypticase Soy Agar plates (TSA; Biomaxima S. A.), then incubated at 34–38 °C for 18–24 h. Each isolate was then profiled biochemically using the API 20E test system according to the recommendations of the supplier (bioMerieux, Marcy l’Etoile, France). The obtained profiles were analysed using version 5.0 of the API 20E database (bioMerieux). All strains identified as *Cronobacter* spp. were additionally verified by amplification and sequencing of 16S rRNA, as described below.

### 2.4 Pigment and capsule production

Production of yellow pigment was assessed following cultivation of the strains on TSA at 30 °C for 48 h, with observations conducted after 24 and 48 h. Extracellular polysaccharide production was evaluated on LA medium with or without 10% (w/v) sucrose, under the same incubation conditions. Yellow pigmentation and mucosity of the bacterial colonies were determined by visual inspection.

### 2.5 Determination of antimicrobial susceptibility

The antimicrobial susceptibility profile of each strain was determined using a standardized Kirby-Bauer disc diffusion method with Mueller-Hinton agar (Oxoid), following the recommendations of the European Committee on Antimicrobial Susceptibility Testing Criteria (EUCAST, 2021). Susceptibility to 12 antimicrobial compounds (Oxoid) recommended for *Enterobacterales* was tested: ampicillin (10 μg), imipenem (10 μg), cefepime (30 μg), cephalexin (30 μg), aztreonam (30 μg), gentamicin (10 μg), amikacin (30 μg), tetracycline (30 μg), chloramphenicol (30 μg), ciprofloxacin (5 μg), trimethoprim-sulfamethoxazole (1.25:23.75 μg) and tigecycline (15 μg). *E. coli* ATCC 25922 was used as a control bacterium. To ensure reproducibility, antimicrobial susceptibility testing was repeated at least twice. Since no official breakpoints have been established for *Cronobacter* spp. by EUCAST (2021), clinical breakpoints for *Enterobacterales* were applied.

### 2.6 Genomic and plasmid DNA isolation

Genomic DNA for use as a template for PCR was extracted from *Cronobacter* spp. cells using a boiling lysis method. One to three colonies from a TSA plate were suspended in 20 μL of lysis buffer (0.25% SDS, 0.05 M NaOH). The suspensions were incubated at 100 °C for 10 min, cooled on ice for 5 min, and then diluted by the addition of 180 μL of cold molecular-grade water. Genomic DNA used for genome sequencing was purified using a Genomic DNA Isolation Kit (DNA Gdańsk). Plasmid DNA was isolated from 1 mL overnight cultures of *Cronobacter* spp. using the alkaline lysis procedure described by (Sambrook and Russell, 2001). Plasmid sizes were estimated by comparing the electrophoretic mobility of the plasmid DNA on a 0.8% agarose gel in TAE buffer with reference plasmids of known size (data not shown).

### 2.7 Molecular characterization of *Cronobacter* strains

Molecular characterization of *Cronobacter* isolates was performed by the amplification and sequencing of the *fusA* and *rpoB* genes. The *fusA* genes were amplified using primers and PCR conditions described in the *Cronobacter* MLST database^1^ (Baldwin et al., 2009; Jolley et al., 2018): amplification with primers F (5′-GAAACCGTATGGCGTCAG-3′) and R (5′-AGAACCGAAGTGCAGACG-3′) followed by sequencing with primers F (5′-GCTGGATGCGGTAATTGA-3′) and R (5′- CCCATACCAGCGATGATG-3′). The *rpoB* genes were amplified and sequenced using primers RpoB_F (5′-AACCAGTTCCGCGTTGGCCTGG-3′) and RpoB_R (5′-CCTGAACAACACGCTCGGA-3′) (Mollet et al., 1997). An additional DNA marker, the sequence of 16S rRNA gene, was used as a confirmatory test of the standard method of *Cronobacter* strain isolation (PN-EN ISO 22964:2017). PCR amplification of the 16S rRNA gene was performed with primers 27f (5′-AGAGTTTGATCCTGGCTCAG-3′) and 1492r (5′-GGTTACCTTGTTACGACTT-3′) (Weisburg et al., 1991). The detection of virulence plasmids in all (26) *Cronobacter* spp. strains was performed by PCR using specific primers F_pVirCro (5’-TCTGAACGGACATCGCATA-3′) and R_pVirCro (5’-TGRCTTTTATGCTGTATTTTCGT-3′) (R = G or A). The detection of circular forms of integrative elements was performed by PCR using the following primer pairs: for IE*Csak_MK10_1 –* IE1MK10F1 (5’-TGGAACGATTCGAGCTTG-3′) and IE1MK10R1 (5’-GCCGTTGGTAGTTACTTC-3′); for IE*Csak_MK10_2* – IE2MK10R1 (5’-CTGTAGACATTCTGCGAG-3′) and IE2MK10F1 (5’-AGTTTATCTCAGGCCAG-3′). Genomic DNA obtained by the boiling lysis method described above was used as the template in all PCRs. Following the amplifications, exonuclease I (ExoI – Thermo Scientific) and alkaline phosphatase (FastAP – Thermo Scientific) were added to the PCR reaction mixtures. After mixing, the tubes were incubated at 37 °C for 15 min, then heated at 85 °C for 15 min to stop the reaction. DNA fragments were then sequenced with an ABI Prism 377 automated sequencer (Applied Biosystems) in the DNA Sequencing and Oligonucleotide Synthesis Laboratory (oligo.pl) at the Institute of Biochemistry and Biophysics, Polish Academy of Sciences.

The obtained nucleotide sequences were trimmed and analyzed using FinchTv 1.4.0 (Geospiza, Inc.; Seattle, WA, United States).^2^ The 16S rRNA gene sequences were compared with sequences deposited in the GenBank database of the National Center for Biotechnology Information^3^ and the Ribosomal Database Project Release 11 (Maidak et al., 1996).^4^ To assign allele numbers the *fusA* and *rpoB* nucleotide sequences were queried against the *Cronobacter* MLST database (Jolley et al., 2018). The *fusA* and *rpoB* gene sequences of the studied isolates were then aligned with the corresponding gene sequences from the following *Cronobacter* strains available in the PubMLST database: *C. dublinensis* LMG 23823^T^, *C. condimenti* LMG 26250^T^, *C. malonaticus* LMG 23826^T^, *C. muytjensii* ATCC 51329^T^, *C. sakazakii* ATCC 29544^T^, *C. turicensis* LMG 23827^T^ and *C. universalis* NCTC 9529^T^, as well as *Citrobacter koseri* FDAARGOS_287^T^, which was used as an outgroup strain in the phylogenetic analyses. The *fusA*-based phylogenetic analysis was performed using the MEGA X tool (version 10.2.4) (Kumar et al., 2018). The tree was constructed using the Neighbor-joining algorithm (Saitou and Nei, 1987) with the Kimura 2 parameter method (Kimura, 1980) and statistical support for internal nodes was determined by 1,000 bootstrap replicates.

### 2.8 Genomic sequencing, sequence assembly and analysis

Genomic DNA was sequenced using both Oxford Nanopore Technologies (ONT) and Illumina platforms. For ONT sequencing, libraries were prepared using mechanical fragmentation (BD Micro-Fine Plus needles) and the Ligation Sequencing Kit with Native Barcoding Kit 96 V14 (SQK-NBD114.96; Oxford Nanopore Technologies). Quality was assessed using the Agilent TapeStation 2,200 with Genomic DNA ScreenTape, and sequencing was performed on a MinION with an R10.4.1 flow cell. For Illumina sequencing, DNA was fragmented using a Covaris S220 ultrasonicator and libraries were prepared with the KAPA HyperPrep Kit and TruSeq DNA UD Indexes. Quality control was performed using the Agilent TapeStation 2,200 using High Sensitivity D1000 reagents. Library quantification and fragment size distribution were assessed by qPCR using the KAPA Library Quantification Kit. Sequencing was performed on an Illumina NovaSeq 6,000 instrument using an S1 Reagent Kit v1.5. Nanopore reads were basecalled with Guppy (Oxford Nanopore Technologies). Illumina reads were quality-filtered with fastp and Nanopore reads were filtered with fastplong (Chen, 2023) to generate two datasets: one for assembly (using gentle filtering) and one for polishing (using more stringent parameters). Residual adapter sequences in Nanopore reads were removed with Porechop_ABI (Bonenfant et al., 2022). Genome assembly was performed using Trycycler (Wick et al., 2021). Nanopore reads were divided into 12 subsets and assembled with three different long-read assemblers: Flye (Kolmogorov et al., 2019), miniasm (Li, 2016) combined with Minipolish (Wick and Holt, 2022) and Raven (Vaser and Šikić, 2021). The resulting assemblies were clustered and manually curated to remove those containing non-circular contigs, low identity, or large size discrepancies. Trycycler modules were used to generate a single consensus assembly. In addition, a short-read-first hybrid assembly was generated using Unicycler (Wick et al., 2017), which produced one extra contig corresponding to plasmid pCS-MK10_P3. This contig was absent from the long-read-first assemblies and was manually incorporated into the Trycycler consensus. The filtered long reads were used to polish the assembly with Medaka (“nanoporetech/medaka,” 2025). Short reads were mapped to the assembly with BWA-MEM2 (Md et al., 2019) and processed with SAMtools (Danecek et al., 2021); the resulting BAM files were used for polishing with Pilon (Walker et al., 2014). Contigs were then rotated using Dnaapler (Bouras et al., 2024) to start at the *dnaA* gene (chromosome) or *rep* gene (plasmids). After rotation, Illumina reads were remapped, and polishing with Pilon was repeated. A final round of polishing was performed with Polypolish (Wick and Holt, 2022). Assembly statistics, including mean sequencing depth, were calculated using SAMtools on reads mapped with minimap2 (Li, 2018) (long reads) and BWA-MEM2 (short reads). Genome annotation was performed using Bakta (Schwengers et al., 2021) and assembly completeness was assessed with BUSCO (Manni et al., 2021) on enterobacteriaceae_odb12 dataset.

Homology searches were conducted using BLASTN and BLASTP tools available on the NCBI website, with default settings (Altschul et al., 1997), and the HHpred server (Söding et al., 2005). Identification of CRISPR spacer sequences targeting plasmid and prophage sequences was performed using concatenated spacer sequences and BLASTN (optimized for somewhat similar sequences). The sequence type of the MK_10 strain was determined using the MLST 2.0 database (Larsen et al., 2012). Comparative genomic analyses were conducted with Proksee (Grant et al., 2023). Prophage sequences were predicted using PHASTEST (PHAge Search Tool with Enhanced Sequence Translation; Wishart et al., 2023), accessed through the Proksee platform^5^ (only intact prophage regions were considered). CRISPR-Cas systems were identified and characterized using CRISPRCasFinder (Couvin et al., 2018). Insertion sequences (IS) were identified and classified through comparative sequence analysis using the ISfinder database (Siguier et al., 2006). A search for integrative and conjugative/mobilizable elements was performed using ICEfinder 3.0 (Wang et al., 2024). The plasmid replication system types were determined using the PlasmidFinder database (Carattoli et al., 2014).

### 2.9 Nucleotide sequence accession numbers

Nucleotide sequences of *Cronobacter* spp. determined in this study were deposited in the GenBank (NCBI) database under the following accession numbers: MW644652–MW644677 (16S rRNA genes) and JBSUQN000000000 (genomic sequences of MK_10). The nucleotide sequence of the insertion sequence IS*Ehe3*a was deposited in the ISfinder database (Siguier et al., 2006).

## 3 Results

### 3.1 Identification of *Cronobacter* spp. in food samples

A total of 251 samples – including raw milk (*n* = 63), powdered milk (*n* = 10), infant formula (*n* = 45), dried herbs and spices (*n* = 110), teas (*n* = 18), and vegetables (*n* = 5) – were analyzed for the presence of *Cronobacter* spp. Based on the characteristic blue colonies observed on chromogenic medium, presumptive *Cronobacter* strains were detected in 26 samples (10.4%). The distribution of positive samples across food categories was as follows: 24 positives in dried herbs and spices (21.8% of all samples in this category), 1 in dried fruit tea, and 1 in raw milk. No *Cronobacter* contamination was detected in powdered milk, infant formula, or vegetables (Table 1). These findings indicate that dried herbs and spices represent the highest-risk food category for *Cronobacter* contamination, underscoring their significant epidemiological relevance in foodborne pathogen surveillance. One isolate per positive sample was further analyzed. Phenotypic analyses using API 20E tests and 16S rRNA gene sequencing confirmed the assignment of all 26 isolates to the genus *Cronobacter* (Iversen et al., 2007).

**Table 1:** Molecular characterization of *Cronobacter* spp. strains isolated from food products.

Strain	Source	Accession number of 16S rRNA*	Molecular characterization		
			16S rRNA	*fusA* [allele number]	*rpoB* [allele number]
MK_1	fresh milk	MW644652	*C. sakazakii*	*C. sakazakii ^[8]^*	*C. sakazakii ^[62]^*
MK_2	herbes de Provence	MW644653	*C. sakazakii*	*C. sakazakii ^[8]^*	*C. sakazakii ^[99]^*
MK_3	black ground pepper	MW644654	*C. turicensis*	*C. turicensis ^[22]^*	*C. turicensis ^[45]^*
MK_4	herby pepper	MW644655	*C. sakazakii*	*C. sakazakii ^[1]^*	*C. sakazakii ^[1]^*
MK_5	basil leaves	MW644656	*C. muytjensii*	*C. muytjensii ^[35]^*	*C. muytjensii ^[57]^*
MK_6	oregano leaves	MW644657	*C. sakazakii*	*C. sakazakii ^[8]^*	*C. sakazakii ^[99]^*
MK_7	spicy pepper	MW644658	*C. muytjensii*	*C. muytjensii ^[25]^*	*C. muytjensii ^[74]^*
MK_8	herby pepper	MW644659	*C. dublinensis*	*C. dublinensis ^[21]^*	*C. dublinensis ^[5]^*
MK_9	herbal tea	MW644660	*C. sakazakii*	*C. sakazakii ^[118]^*	*C. sakazakii ^[62]^*
**MK_10**	**oregano leaves**	**MW644661**	***C. sak./C.mal.***	***C. sakazakii*** ^***[8]***^	***C. sakazakii*** ^***[21]***^
MK_11	herbes de Provence	MW644662	*C. sak./C.mal.*	*C. malonaticus ^[7]^*	*C. malonaticus ^[18]^*
MK_12	tarragon	MW644663	*C. sakazakii*	*C. sakazakii ^[17]^*	*C. sakazakii ^[59]^*
MK_13	marjoram leaves	MW644664	*C. muytjensii*	*C. muytjensii ^[35]^*	*C. muytjensii ^[74]^*
MK_14	basil leaves	MW644665	*C. turicensis*	*C. turicensis ^[39]^*	*C. turicensis ^[103]^*
MK_15	sweet pepper	MW644666	*C. sakazakii*	*C. sakazakii ^[14]^*	*C. sakazakii ^[94]^*
MK_16	sweet pepper	MW644667	*C. muytjensii*	*C. muytjensii ^[177]^*	*C. muytjensii ^[74]^*
MK_18	sweet pepper	MW644668	*C. sakazakii*	*C. sakazakii ^[18]^*	*C. sakazakii ^[22]^*
MK_19	herbes de Provence	MW644669	*C. sak./C.mal.*	*C. malonaticus ^[13]^*	*C. malonaticus ^[18]^*
MK_20	herbes de Provence	MW644670	*C. sakazakii*	*C. sakazakii ^[1]^*	*C. sakazakii ^[1]^*
MK_25	herby pepper	MW644671	*C. sakazakii*	*C. sakazakii ^[1]^*	*C. sakazakii ^[1]^*
MK_26	basil leaves	MW644672	*C. sakazakii*	*C. sakazakii ^[1]^*	*C. sakazakii ^[1]^*
MK_28	sweet pepper	MW644673	*C. sakazakii*	*C. sakazakii ^[11]^*	*C. sakazakii ^[23]^*
MK_29	basil leaves	MW644674	*C. muytjensii*	*C. muytjensii ^[35]^*	*C. muytjensii ^[74]^*
MK_31	marjoram leaves	MW644675	*C. muytjensii*	*C. muytjensii ^[25]^*	*C. muytjensii ^[74]^*
MK_32	basil leaves	MW644676	*C. muytjensii*	*C. muytjensii ^[25]^*	*C. muytjensii ^[74]^*
MK_37	herbes de Provence	MW644677	*C. turicensis*	*C. turicensis ^[39]^*	*C. turicensis ^[116]^*

*Accession numbers of the 16S rRNA nucleotide sequences of *Cronobacter* spp. deposited in the GenBank (NCBI) database (https://ncbi.nlm.nih.gov). The strain used for the genomic analysis is shown in bold.

### 3.2 Molecular classification of *Cronobacter* strains

**Figure 1:**
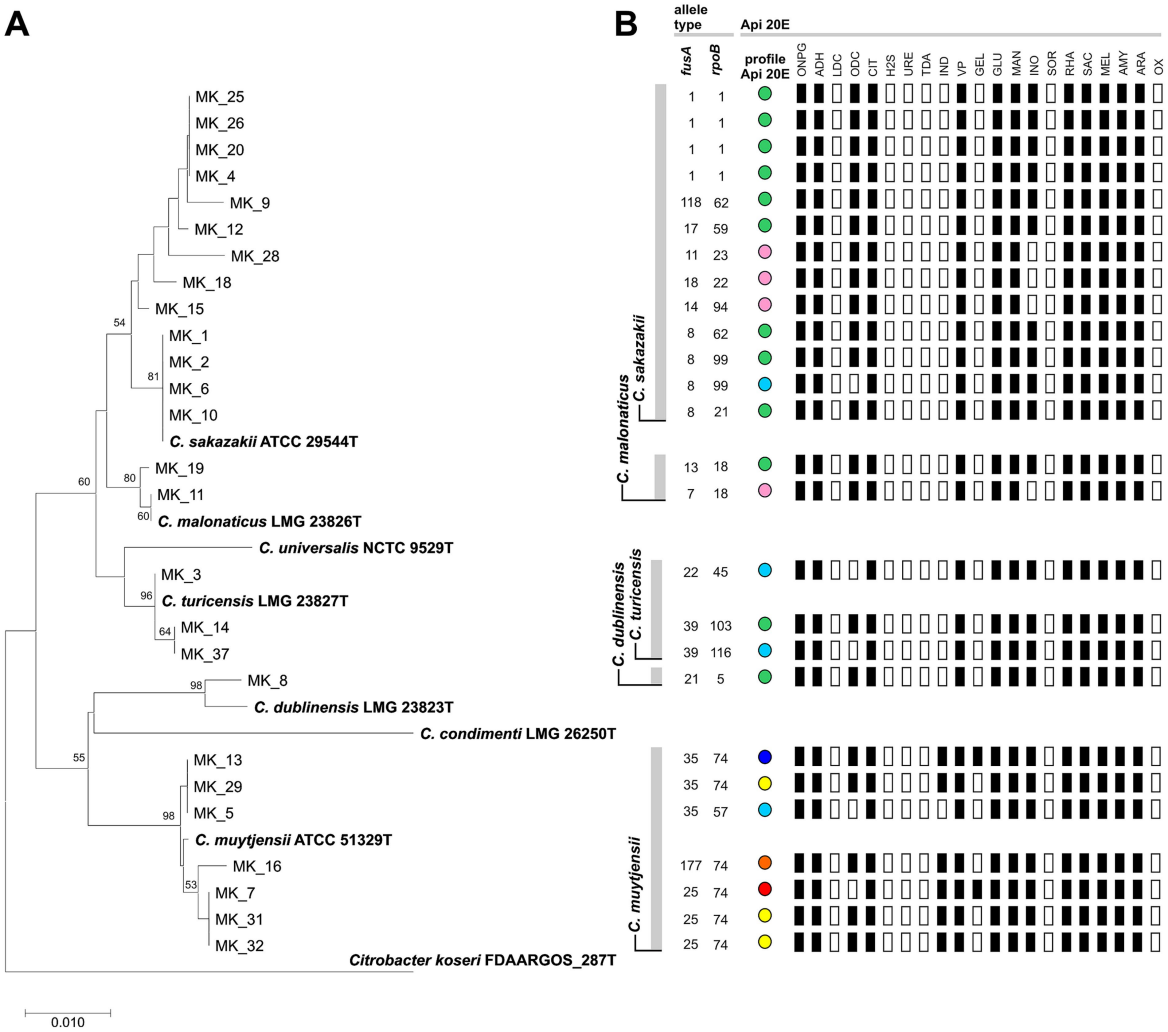
Phylogenetic, molecular and biochemical characterization of 26 *Cronobacter* spp. strains isolated from food products. **(A)** Phylogenetic analysis on the basis of *fusA* gene sequences of the isolated strains and reference type strains of all *Cronobacter* species (the *fusA* sequence of *Citrobacter koseri* FDAARGOS_287^T^ was used as an outgroup). The phylogenetic tree was constructed using the neighbor-joining algorithm with the Kimura 2 parameter method and 1,000 bootstrap replicates (values above 50% are shown) (MEGA X; version 10.2.4). **(B)** Molecular and biochemical characterization of the strains – classification of *fusA* and *rpoB* alleles and biochemical profile. The colored dots indicate different biochemical profiles, clustering the *Cronobacter* strains based on their API 20E test results. API 20E tests (bioMerieux): ONPG, *β*-galactosidase activity; ADH, arginine dihydrolase activity; LDC, lysine decarboxylase activity; ODC, ornithine decarboxylase activity; CIT, utilization of citrate; H_2_S, production of hydrogen sulfide; URE, urease activity; TDA, tryptophan deaminase activity; IND, indole production; VP, the Voges-Proskauer test for the detection of acetoin; GEL, production of gelatinase; GLU, fermentation of glucose; MAN, fermentation of mannose; INO, fermentation of inositol; SOR, fermentation of sorbitol; RHA, fermentation of rhamnose; SAC, fermentation of sucrose; MEL, fermentation of melibiose; AMY, fermentation of amygdalin; ARA, fermentation of arabinose; OX, cytochrome oxidase activity.

The 16S rRNA gene does not always permit identification of *Cronobacter* strains at the species level due to the high sequence similarity among species (Iversen et al., 2004; Forsythe, 2018). Therefore, molecular classification of *Cronobacter* isolates was performed using 2 alternative DNA markers: the *fusA* and *rpoB* genes. We identified 15 distinct *fusA* allele profiles and 15 *rpoB* allele profiles (listed in Figure 1B), which enabled the assignment of each isolate to a specific *Cronobacter* species (Table 1; Figure 1). These results were consistent with the topology of a phylogenetic tree constructed using *fusA* gene nucleotide sequences of the analyzed isolates and *Cronobacter* spp. reference strains (Figure 1A). The most prevalent species recovered from the samples were *C. sakazakii* (*n* = 13; 50%) and *C. muytjensii* (*n* = 7; 26.9%). The remaining isolates were identified as *C. turicensis* (*n* = 3; 11.5%), *C. malonaticus* (*n* = 2; 7.7%) and *C. dublinensis* (*n* = 1; 3.8%).

### 3.3 Biochemical profiles and resistance phenotypes of *Cronobacter* isolates

Seven different biochemical profiles were identified among the *Cronobacter* isolates using API 20E tests. Overall, the isolates exhibited highly similar biochemical features, differing only in a few specific traits: (i) ornithine decarboxylation (ODC) – this activity was absent in five strains (*C. muytjensii* MK_5, MK_7, *C. sakazakii* MK_6, *C. turicensis* MK_3, MK_37), (ii) gelatin liquefaction (GEL) – positive results were observed only for two *C. muytjensii* strains (MK_7, MK_13), (iii) inositol fermentation (INO) – four strains lacked this ability (*C. sakazakii* MK_15, MK_18, MK_28 and *C. malonaticus* MK_11) and (iv) indole production (IND) – this trait was detected exclusively in *C. muytjensii* strains, with the exception of strain MK_5 (Figure 1B). Indole production has been previously reported as a distinguishing feature of *C. muytjensii* (Jackson and Forsythe, 2016).

The production of a characteristic yellow carotenoid pigment was observed in all tested *Cronobacter* isolates grown on TSA medium. Extracellular polysaccharide production was observed in 25/26 isolates (strong capsule forming) grown on sucrose-containing LA medium, with the exception of *C. malonaticus* MK_11.

The antimicrobial susceptibility of the *Cronobacter* isolates was tested using a panel of 12 antibacterial agents representing a broad spectrum of the antibiotics commonly used against *Enterobacterales* infections: ampicillin, imipenem, cefepime, cephalexin, aztreonam, gentamicin, amikacin, tetracycline, chloramphenicol, ciprofloxacin, trimethoprim-sulfamethoxazole and tigecycline. All strains were susceptible to all of the tested compounds (data not shown).

### 3.4 Plasmid profiles

**Figure 2:**
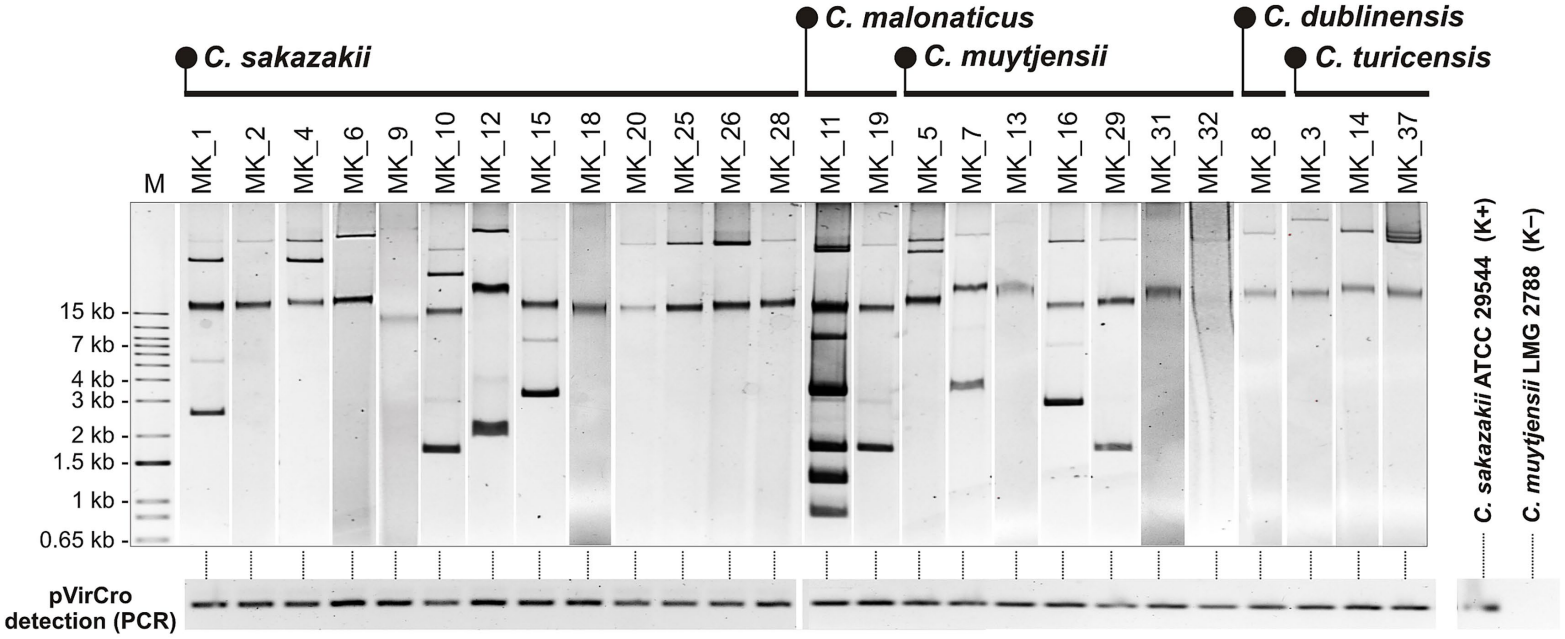
Plasmid profiles of tested *Cronobacter* spp. isolates (upper panel) and detection of pVirCro-type plasmids using PCR (lower panel). Extrachromosomal DNA was extracted using the alkaline lysis method and separated by agarose gel electrophoresis. M – DNA ladder (1 Kb Plus DNA Ladder, Invitrogen). In the PCR-based detection of pVirCro plasmids, *C. sakazakii* ATCC 29544 (harboring a pVirCro-type plasmid) and *C. muytjensii* LMG 2788 (plasmid-less strain; confirmed by WGS sequencing; unpublished data) were used as positive and negative controls, respectively.

The 26 *Cronobacter* isolates were screened for the presence of plasmids. As shown in Figure 2, each strain contained at least one extrachromosomal replicon, and some carried multiple replicons of varying sizes. Previous studies have demonstrated that the vast majority of *Cronobacter* spp. strains carry characteristic, highly similar IncFIB plasmids, commonly referred to as virulence plasmids related to pESA3 (131 kb) of *C. sakazakii* (designated in this study as pVirCro), as they encode potential virulence factors, including two iron (III) acquisition systems and components of Type VI secretion systems (T6SS) (Jang et al., 2020; Franco et al., 2011; Grim et al., 2012).

To confirm the presence of pVirCro plasmids in the analyzed strains, we designed specific oligonucleotide primers for PCR by aligning the complete nucleotide sequences of pVirCro plasmids from various *Cronobacter* species available in the GenBank database. Based on the conserved regions, a primer pair (F_pVirCro and R_pVirCro) was designed, enabling the amplification of a 321-bp pVirCro-specific DNA fragment, encompassing parts the replication and partitioning regions of these plasmids. The specificity of this PCR was confirmed using genomic DNA of fully sequenced *Cronobacter* spp. strains (obtained from microbial culture collections) which contain or lack pVirCro. Subsequent application of this PCR revealed that all *Cronobacter* isolates carried pVirCro-type plasmids (Figure 2).

### 3.5 Genome characterization of the *Cronobacter sakazakii* MK_10 strain

The strain MK_10 was selected for genomic analysis as a representative of the most prevalent species in the collected strain pool, *C. sakazakii*, known for its high pathogenic potential. In addition, the strain exhibited a unique plasmid profile (Figure 2). The complete nucleotide sequence of the MK_10 genome revealed a circular chromosome (4,286,193 bp; 3,908 coding DNA sequences, CDSs) and three extrachromosomal replicons (ECRs), designated pCS-MK10_P1 (114,084 bp; 107 CDSs), pCS-MK10_P2 (45,949 bp; 49 CDSs) and pCS-MK10_P3 (2733 bp) (3 CDSs). According to the sequence annotation, MK_10 contains 10 pseudogenes and encodes 83 tRNAs, 7 rRNA gene clusters, and 46 predicted non-coding regulatory RNAs. The G + C content of the three plasmid sequences is 57% (comparable to the chromosomal G + C content of 56.9%), 49.1% and 48.3%, respectively. Multilocus sequence typing (MLST 2.0) classified the isolate as a sequence type 31 (ST31).

**Figure 3:**
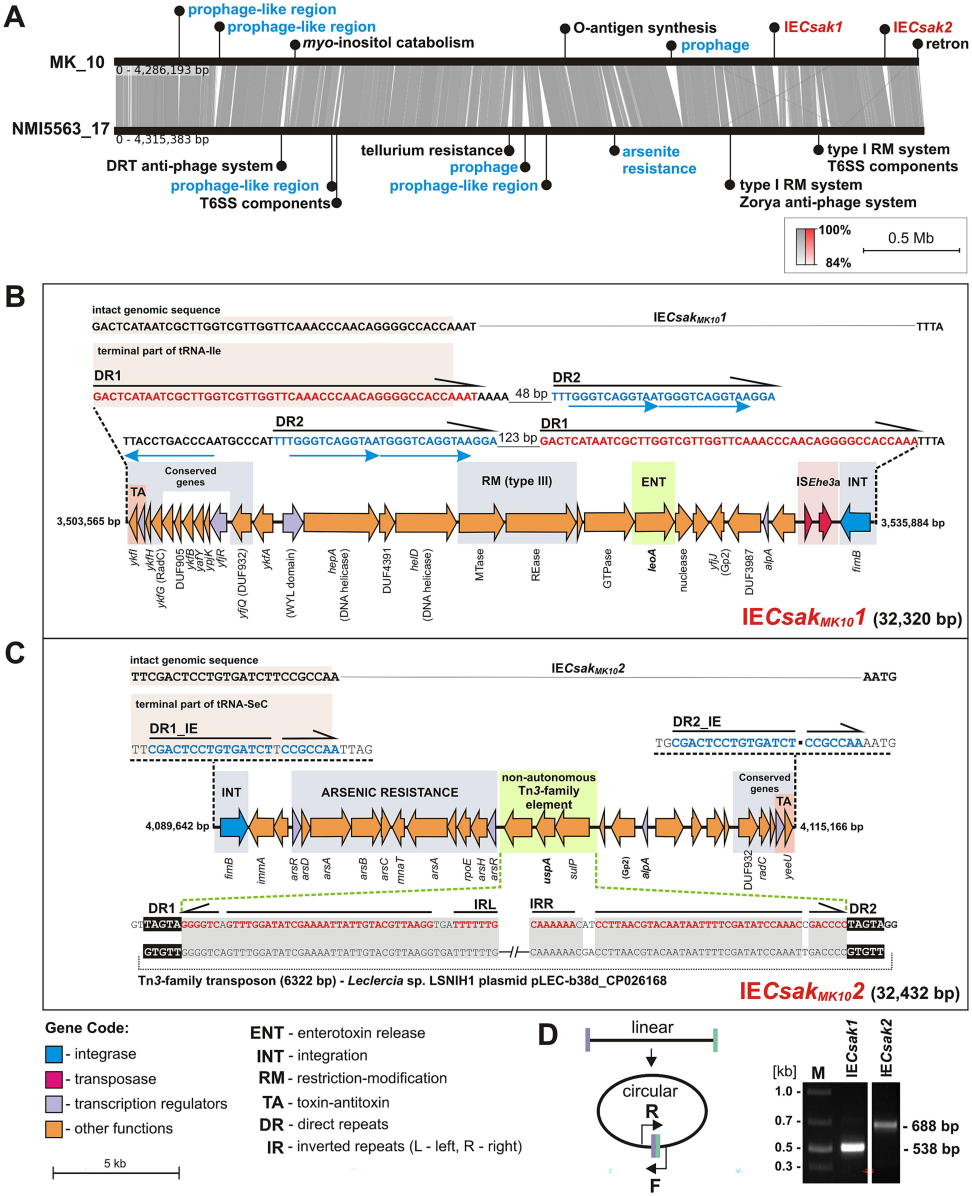
The influence of prophages and other integrative elements on the architecture of the *C. sakazakii* MK_10 and NMI5563_17 chromosomes. **(A)** Whole-genome synteny analysis highlighting selected chromosomal DNA regions or predicted functions unique to each strain. Gray shading indicates regions of sequence identity. Notable regions of difference are indicated with descriptions in blue and red denoting predicted mobile genetic elements. DRT – defense-associated reverse transcriptase system, IE – integrative element, RM – restriction-modification system. **(B,C)** Integrative elements IE*Csak_MK10_1* and IE*Csak_MK10_2* identified in the MK_10 chromosome. The numbers at both ends of the elements indicate the nucleotide position within the MK_10 chromosome sequence. Genes conserved in both elements are indicated. Genes/elements with the potential to contribute to pathogenicity are highlighted in green. Attachment sites of IE*Csak_MK10_1* and IE*Csak_MK10_2* are indicated. **(D)** Detection of circular forms of IE*Csak_MK10_1* and IE*Csak_MK10_2*. The right and left attachment sites are indicated in violet and green, respectively. F, R – forward and reverse primers (IE1MK10F1 and IE1MK10R1 – for IE*Csak_MK10_1*; IE2MK10R1 and IE2MK10F1 – for IE*Csak_MK10_2*). Right panel – electrophoretic detection of amplicons.

We performed a comparative genomic analysis of MK_10 and *C. sakazakii* NMI5563_17, a strain previously identified as the cause of a fatal infection in a premature infant at Wroclaw Medical University (the first completely sequenced *C. sakazakii* strain isolated in Poland; Lachowska et al., 2021). As shown in Figure 3A, the chromosomes of these strains display synteny and a high level of sequence conservation. Structural variations are limited to insertions and deletions of DNA fragments of variable length, with no evidence of inversions or translocations. Many of these regions of difference contain prophage-like elements and intact prophages (Figure 3A), confirming the key role of bacteriophages in shaping the chromosome structure of these bacteria (Zeng et al., 2017).

MK_10 and NMI5563_17 share the same set of genes previously reported to be associated with pathogenesis (Liu et al., 2024), including those likely to be involved in cell adherence and invasion, immune evasion and cell motility, highlighting the pathogenetic potential of the environmental MK_10 strain. Each strain also harbors a unique pool of genes – 332 (MK_10) and 310 (NMI5563_17) (Supplementary Table S1). In the case of MK_10, this includes 75 genes encoding hypothetical proteins, numerous phage-related genes, as well as genes related to inositol catabolism (e.g., *myo*-inositol catabolism protein LolC) and O-antigen modification (e.g., genes encoding O-antigen polymerase, flippase and several glycosyltransferases; Figure 3A). In contrast, NMI5563_17 contains unique predicted anti-phage systems (of the DTR and Zorja types), a tellurium resistance gene cluster, type I restriction–modification systems, and additional components of the type VI secretion system (T6SS) (Figure 3A). A complete list of the strain-specific genes is provided in Supplementary Table S1.

A unique feature of MK_10 is the presence of genes encoding an RNA-directed DNA polymerase and an effector protein, both characteristic of the St85 retron family (Millman et al., 2020). The presence of predicted DNA regions corresponding to the retron msrRNA and the STnc400 small RNA in their immediate vicinity strongly suggests the existence of a complete retron (nt position: 4,278,348–4,280,749). Identical sequences are also present in whole-genome sequencing (WGS) contigs of other *C. sakazakii* strains, including CE56 C301 isolated from PIF in China (GenBank: SWWW01000012) and CFSAN135117 isolated from seeds in the USA (GenBank: ABPDAV010000011). Highly related sequences (95–99% sequence identity) were also identified (BLASTN) in the chromosomes of *Citrobacter freundii* RHBSTW-00965 (GenBank: CP056232), several *E. coli* and *Klebsiella pneumoniae* strains, as well as numerous *Salmonella enterica* isolates.

### 3.6 Prophages and CRISPR-Cas immunity system

Analysis using the PHASTEST tool revealed that the MK_10 chromosome contains a single intact prophage (with a G + C content of 50%) located in close proximity to the arginine tRNA gene (Figure 3A). This prophage is predicted to encode 63 proteins with matches to known phage proteins, as well as 14 hypothetical proteins. It is most similar to the temperate *Cronobacter* phage ENT47670 (GenBank: NC_019927), which shares 21 proteins with the MK_10 prophage. Although identical prophages were not found in fully assembled genomes, we detected them, integrated at the same genomic locations, in WGS contigs of several *C. sakazakii* strains, including CE56 isolated from PIF in China (GenBank: SWWW01000014) and CFSAN135117 isolated from seeds in the United States (GenBank: ABPDAV010000002).

**Figure 4:**
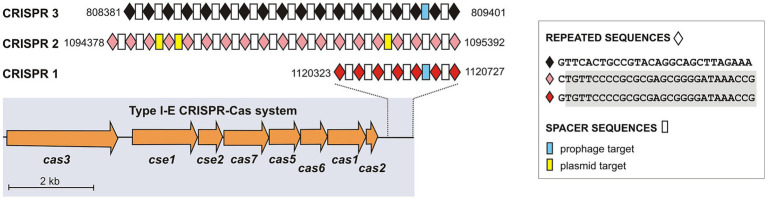
Genetic organization of the CRISPR–Cas locus in the chromosome of *C. sakazakii* MK_10. The genome harbors three CRISPR *loci*, two of which (CRISPR_2 and CRISPR_3) are not associated with *cas* genes. Identical repeat sequences shared between CRISPR_1 and CRISPR_2 are highlighted in gray. Spacer sequences targeting plasmid or prophage sequences are indicated accordingly.

The MK_10 chromosome also carries a CRISPR-Cas anti-phage immune system composed of eight *cas* genes, characteristic of subtype I-E, and three CRISPR arrays (CRISPR_1–3) consisting of short direct repeats (DRs) interspaced with variable spacer sequences acquired from invading DNA (Figure 4). The *cas* gene cluster, which encodes effectors of the immune response, includes *cas3*, *cse1*, *cse2*, *cas7*, *cas5*, *cas6*, *cas1* and *cas2* (nucleotide, nt position: 1,111,386–1,120,226), and is associated with the CRISPR_1 array. The two other solo CRISPR loci (CRISPR_2 and CRISPR_3) are located approx. 26 kb and 310 kb upstream of CRISPR_1, respectively. Both CRISPR_1 and CRISPR_2 contain highly conserved 29-bp DRs with nearly identical consensus sequences differing only at the first nt position (Figure 4). These repeats are interspaced with 6 and 18 spacer sequences, respectively. In contrast, the CRISPR_3 locus possesses DRs that differ in both length (28 bp) and sequence, and are separated by 17 spacers. Altogether, the CRISPR loci of MK_10 contain a total of 41 unique spacers, none of which are present in the CRISPR arrays of strain NMI5563_17. Most of them do not match any genomic sequences currently found in the GenBank database (BLASTN), however several show significant similarities to sequences of phage or plasmid origin. Notably, three spacer sequences from CRISPR_2 (Figure 4) exhibit 100% identity to the same replicon – the conjugative plasmid pGK1025B_3 (46,528 bp) from *C. sakazakii* strain MOD1-GK1025B (GenBank: CP078109), which was isolated from a PIF-manufacturing setting in Germany (Negrete et al., 2022). These sequence matches occur within the coding regions for a VirB4 family type IV conjugal transfer ATPase, a type II restriction endonuclease of the Mrr-cat superfamily, and a DGQHR domain-containing protein of unknown function. The first of the three aforementioned spacers also perfectly matches sequences in several other plasmids encoding related VirB4 ATPases, including pNK_H10_003.2 of *K. pneumoniae* NK_H10_003 (GenBank: CP152965), pd1-37_C of *E. coli* d1-37 (GenBank: AP040920), and an unnamed plasmid of *Siccibacter colletis* YSD YN2 (GenBank: CP074353).

Two other spacers show sequence similarity (with two mismatches) to different prophages. One of them (CRISPR_3) matches a terminase gene in an intact prophage (53.7 kb) (related to *Escherichia* phage HK639; GenBank: NC_016158) (PHASTEST), integrated within the chromosome of *C. freundii* FDAARGOS_549 (clinical isolate; USA) (GenBank: CP033744). The second spacer (CRISPR_1) matches a gene encoding a phage tail fiber domain-containing protein within an intact prophage (related to *Salmonella* phage SPN1S; GenBank: NC_016761) (PHASTEST), located in the chromosome of *E. coli* ET846 isolated from a frog farm in China (GenBank: CP100895).

Comparative analysis using the NCBI Core Nucleotide databases revealed that among the 41 identified spacer sequences only 2 (from CRISPR_2) were detected (100% identity) in the CRISPR arrays of several *C. sakazakii* strains. In contrast, similar BLASTN searches against the WGS contigs database identified numerous *C. sakazakii* strains harboring CRISPR arrays very similar to those of strain MK_10, including *C. sakazakii* CFSAN135046 isolated from grain in the USA (GenBank: ABPDPK010000002). This confirms and highlights the usefulness of CRISPR sequences and patterns for typing of closely related *Cronobacter* spp. strains and tracking their dissemination pathways (Zeng et al., 2020).

### 3.7 Transposable and integrative elements

Within the MK_10 genome we identified a single insertion sequence (IS), comprising two ORFs (KODCJN_03334, KODCJN_03335) encoding a putative fusion transposase (382 aa) (Figure 3B). The predicted IS element is 1,229 bp in length (nt position: 3,532,844–3,534,072) and is flanked by 3-bp DRs (5′-GTA-3′; Figure 3B). It shares 96% nucleotide sequence identity with IS*Ehe3* (IS*51* group; IS*3* family) of *Pantoea agglomerans* plasmid pPATH, supporting its classification as an isoform of this insertion sequence, designated IS*Ehe3*a (ISfinder). BLAST searches revealed the presence of highly similar ISs (> 99% nucleotide identity), often in multiple copies, within the genomes (chromosomes and plasmids) of various enteric bacteria, including *C. freundii*, *Enterobacter* spp. and *Klebsiella* spp.

Analysis using the ICEfinder tool did not detect any integrative and conjugative or mobilizable elements (ICEs or IMEs) in the MK_10 genome. This is consistent with the absence of predicted relaxase genes typically associated with such elements. However, within two unique genomic regions of MK_10 (absent in NMI5563_17; Figure 3A), integrase genes adjacent to tRNA genes were identified in an arrangement characteristic of many integrative elements. Detailed inspection of these regions enabled the identification of putative left and right attachment sites flanking the predicted elements – two perfect or near-perfect DRs covering the terminal parts of the tRNA genes (for isoleucine and selenocysteine; Figures 3B,C). These predictions were confirmed by PCR with outward-facing primers (Figure 3D), which demonstrated that these elements, designated here as IE*Csak_MK10_1* and IE*Csak_MK10_2* (IE, integrative elements), can excise from their genomic locations and may exist in an autonomous circular form containing one of the DRs. The termini of IE*Csak_MK10_1* exhibit a more complex structure, as they contain additional (internal) repeated sequences (DR2) with shorter repeat motifs (5′-TGGGTCAGGTAA-3′), which may potentially be involved in regulation of the recombination process (Figure 3B).

Both elements encode tyrosine-type integrases of the FimB family; however, these proteins lack significant amino acid (aa) sequence identity. They also carry, at one terminus, several related genes encoding a type IV toxin-antitoxin (TA) system of the CbeA/CbeT family, plus a DUF987-containing protein, RadC-family protein and DUF932-containing protein (the most highly conserved at the aa level; 88% identity; Figures 3B,C) – an arrangement previously observed in so-called cryptic prophages, first identified in the *Escherichia coli* K-12 genome (Blattner et al., 1997). Indeed, IE*Csak_MK10_1* also contains a few other genes that are found in the cryptic prophage CP4-6 (Figure 3B), suggesting a common origin of these elements.

The cargo genes of IE*Csak_MK10_1* (32,320 bp) and IE*Csak_MK10_2* (32,432 bp) are shown in Figures 3B,C. Besides hypothetical or uncharacterized proteins, IE*Csak_MK10_1* encodes several proteins associated with nucleic acid recombination and repair. These include a putative nuclease of the PD-(D/E)XK superfamily, a type III restriction-modification system endonuclease (REase), a site-specific adenine DNA methyltransferase (MTase) of the Mod superfamily, DNA helicase IV, a SNF2 family helicase, and a RadC family DNA repair protein. Of particular interest is the presence of a gene encoding a putative LeoA (heat-labile enterotoxin output A) family dynamin-like GTPase (Figure 3B), since a related protein contributes to enterotoxin release in pathogenic *E. coli* strains (Brown and Hardwidge, 2007).

IE*Csak_MK10_2* carries a large arsenic resistance gene cluster (ARS), encoding two putative ArsR family transcriptional regulators, the arsenate reductase ArsC (catalyzes the reduction of arsenate to arsenite), the ATPase ArsA, the membrane transporter ArsB, and the metallochaperone ArsD – together forming the arsenite efflux system (Fekih et al., 2018). In addition, the gene cluster encodes ArsH, which detoxifies organic arsenic compounds (Yang and Rosen, 2016), as well as a GNAT family N-acetyltransferase and RNA polymerase sigma-70 subunit (Figure 3C).

Interestingly, IE*Csak_MK10_2*, in its core region, contains two long (49 bp) imperfect inverted repeats, homologous to the TIRs of a widespread group of Tn*3*-family transposons, including an unnamed transposon occurring in *Leclercia* sp. LSNIH1 (Figure 3C). Instead of transposase and resolvase genes, these TIRs flank three genes not involved in transposition, encoding two transporters belonging to the MFS (Yan, 2013) and SulP families (Kertesz, 2001), and a protein with a domain characteristic of universal stress proteins (USP), providing adaptation to environmental stressors(Luo et al., 2023). This predicted element, with a structure characteristic of non-autonomous transposons, is flanked by 5-bp DRs (a length typical for Tn*3*-family transposons), being a genetic scar of a past transposition event (Figure 3C).

BLAST analysis of available *Cronobacter* spp. sequences identified only one element related to IE*Csak_MK10_1* (differing by just two nucleotides) in *C. sakazakii* strain MOD1_LR753, isolated from honey powder in the United States (contig_13; GenBank: PTOS01000021). Genes encoding LeoA homologs were also detected in two other strains: *C. sakazakii* MOD1-E266 isolated from a milk powder processing plant in the Netherlands (GenBank: MDI7680032) (98% aa identity with MK_10 LeoA) and *C. dublinensis* CFSAN136333 (GenBank: EOC1350322), an environmental isolate (59% aa identity). In contrast, more IE*Csak_MK10_1*-like elements were detected in the chromosomes of various *Enterobacter* spp., including *E. kobei* Ek72 (GenBank: CP088229) and *E. hormaechei* RHBSTW-00086 (GenBank: CP058157) – however, none of these elements encode a LeoA family dynamin-like GTPase (data not shown).

Elements identical or nearly identical to IE*Csak_MK10_2* were identified (BLASTN) in three completely sequenced genomes of *E. hormachei* strains A32614 (GenBank: CP181762), 189 (GenBank: CP047965) and B19446 (GenBank: CP144716). However, closely related elements were found in numerous *Enterobacter* strains, lacking the non-autonomous Tn*3*-family transposon in most cases. Highly related elements were also identified within sequence contigs of multiple *C. sakazakii* strains, which may reflect their adaptive significance.

### 3.8 Extrachromosomal replicons

**Figure 5:**
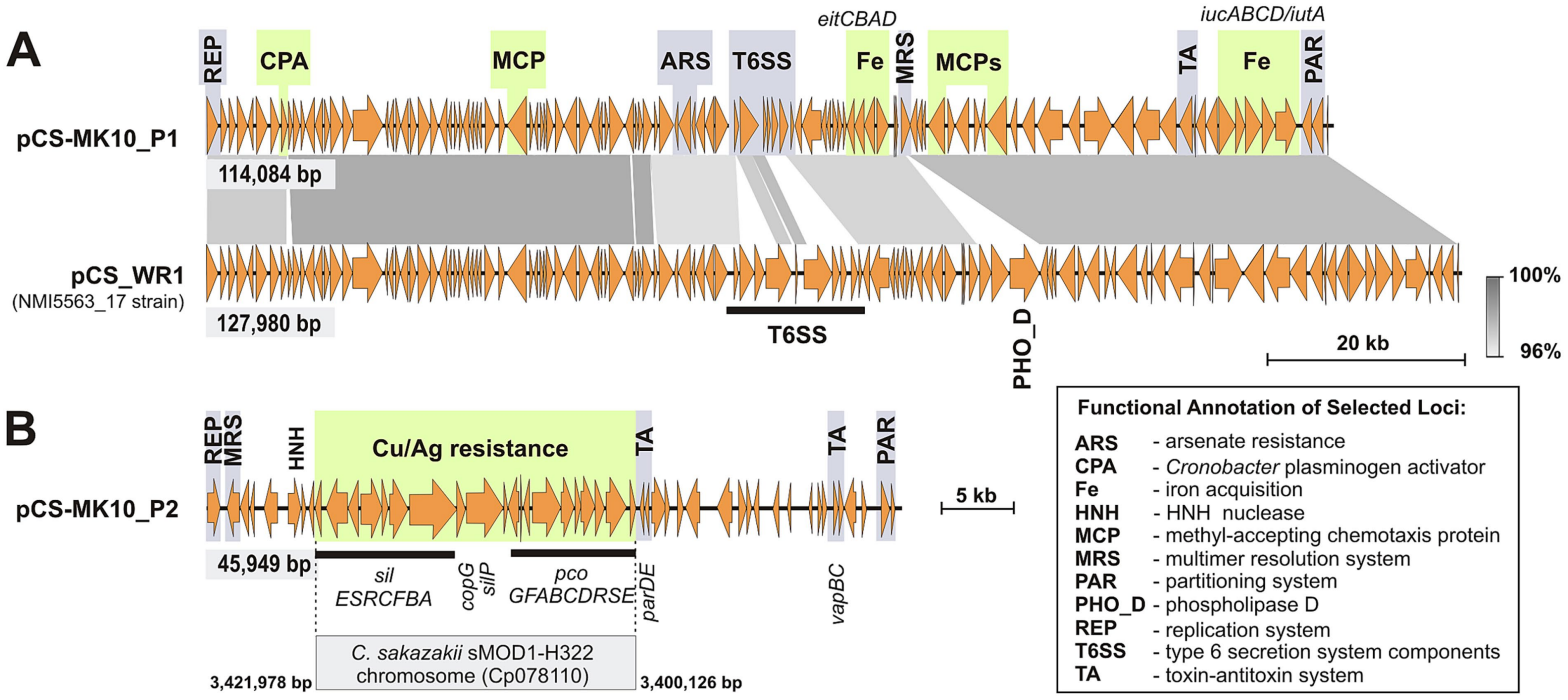
Genetic organization of IncFIB plasmids of *C. sakazakii* MK_10. Genes with predicted functions potentially contributing to pathogenicity are marked in green. **(A)** Comparison of major structural features of two related pVirCro plasmids – pCS-MK10_P1 of MK_10 and pCS_WR1 of *C. sakazakii* NMI5563_17 (GenBank: NZ_MT759836). Gray shading indicates regions of sequence identity. **(B)** Genetic modules identified in pCS-MK10_P2, with copper/silver resistance gene clusters highlighted (conserved also, e.g., in the chromosome of *C. sakazakii* sMOD1-H322; GenBank: Cp078110).

The MK_10 plasmids pCS-MK10_P1 and pCS-MK10_P2 exhibit a high level of sequence identity to the *C. sakazakii* NMI5563_17 plasmids pCS-WR1 and pCS-WR2, respectively. pCS-MK10_P1 encodes a replication initiation protein of the IncFIB type (PlasmidFinder). It also carries (i-ii) a type Ia partitioning system (PAR) and a *hipAB* family toxin-antitoxin system (TA), which contribute to plasmid stabilization at the segregational and post-segregational levels, respectively, and (iii) a tyrosine integrase gene – a potential component of a multimer resolution system (MRS) (Figure 5A).

pCS-MK10_P1 and pCS-WR1 (127,980 bp) of *C. sakazakii* NMI5563_17 (GenBank: NZ_MT759836) exhibit the typical architecture of pVirCro plasmids, characterized by synteny and a high level of sequence conservation (Figure 5A). Both replicons harbor, among other features, two iron (III) acquisition systems (*eitCBAD* – ABC heme transporter, and *iucABCD/iutA* – encoding cronobactin, an aerobactin-like siderophore) and the *Cronobacter* plasminogen activator gene (*cpa*), which has a documented role in pathogenesis (Franco et al., 2011; Singh et al., 2015). In addition, they encode three putative methyl-accepting chemotaxis proteins (MCPs), which may be involved in chemotactic signal transduction and could contribute to cell survival and virulence (Salah Ud-Din and Roujeinikova, 2017), as well as ArsRBC proteins possibly involved in arsenate resistance (ArsR – regulatory protein, ArsC arsenate reductase that converts arsenate to the less toxic arsenite, ArsB – arsenite transporter; Fekih et al., 2018; Figure 5A). As shown in Figure 5A, the main structural differences between the two plasmids involve several genes encoding components of the T6SS and a phospholipase D gene, which are both absent in pCS-MK10_P1. Comparative sequence analysis (BLASTN) revealed that sequences nearly identical to pCS-MK10_P1 occur in WGS contigs of several *C. sakazakii* strains isolated in the USA, including strain CFSAN135037, recovered from grain (GenBank: ABPDLH010000014), and an environmental monitoring isolate 2012-05-20 (GenBank: ABPXBP010000009). Both contigs are of the same length as pCS-MK10_P1 (114,084 bp) and differ by only 17 and 24 single-nucleotide mismatches, respectively.

pCS-MK10_P2 also carries an IncFIB-type replication system (PlasmidFinder), along with predicted PAR and MRS systems, as well as two TA systems belonging to the ParED and VapBC families (Figure 5B). This plasmid is nearly identical to pCS-WR2 of the strain NMI5563_17 (GenBank: NZ_MT759837), differing by only four single-nucleotide mismatches (BLASTN). The core region of the plasmid contains a conserved genetic module comprising the *sil* (silver sensing and efflux system) and *pco* (copper resistance) gene clusters. Related metal resistance islands are widely distributed among *Enterobacteriaceae* and may provide additional fitness benefits to pathogenic strains (Osman et al., 2010; Achard et al., 2012). Plasmid pCS-MK10_P2, in addition to pCS-WR2, shows sequence similarity to two other related, albeit larger, replicons: pCS3 (53,383 bp) of *C. sakazakii* NCTC 8155 (GenBank: CP012256), isolated from milk in the UK (Moine et al., 2016) and pSP291-2 (52,134 bp) of *C. sakazakii* SP291 (GenBank: CP004093), recovered from a PIF production facility in Ireland (Power et al., 2013).

The smallest MK_10 plasmid, pCS-MK10_P3 (2733 bp), is a cryptic replicon. Its predicted replication system shows an arrangement typical for ColE1-type plasmids, in which replication initiation occurs via an RNA-mediated mechanism (Cesareni et al., 1991). Comparison with the Rfam database identified two plasmid DNA regions encoding sequences homologous to the *E. coli* preprimer RNAII (551 nt; nt positions 1999–2549) and a short antisense RNAI (99 nt; nt positions 2444–2542), which is complementary to the 5′ region of RNAII (data not shown). In ColE1-like plasmids RNAI acts as a negative regulator of replication initiation (Freudenau et al., 2015). Interestingly, no sequences fully identical to pCS-MK10_P3 were detected. The closest matches covered approximately 72% of the plasmid sequence, with 97–99% nucleotide identity, corresponding to several mobilizable plasmids, including pSP291_3 (4222 bp) of the aforementioned *C. sakazakii* strain SP291 (GenBank: CP004094), pRHBSTW_00449_3 (4426 bp) of *Citrobacter freundii* (GenBank: CP056507), and pRHBSTW_00610_5 (4426 bp) of *K. pneumoniae* (GenBank: CP056389).

## 4 Discussion

We investigated the prevalence of bacteria of the genus *Cronobacter* in various types of food products, including dried herbs and spices. Among 251 analyzed samples we identified 26 strains representing five *Cronobacter* species. The most frequently identified species were *C. sakazakii* (13) and *C. muytjensii* (7), both exhibiting considerable diversity in their *fusA* and *rpoB* alleles. Although *Cronobacter* spp. are sensitive to high temperatures, their presence in dried herbs and spices (in 24 tested samples), which are commonly added to foods without subsequent thermal processing, may pose a potential health risk, particularly for elderly or immunocompromised individuals. This risk is further exacerbated by their ability to persist for extended periods in low-water-activity environments (Tall et al., 2019). Our findings are consistent with previous studies reporting even higher contamination rates in dried herbs and spices: e.g. 26.9% in Switzerland (Baumgartner et al., 2009), 28.3% in the Czech Republic (Hochel et al., 2012), 32.7% in the United Kingdom (Iversen and Forsythe, 2003), 36.7% in Brazil (Brandão et al., 2017), 38.8% in Jordan (Jaradat et al., 2009) and 57.1% in China (Liu et al., 2018). This indicates a global problem that could be mitigated by applying final irradiation or heat treatment to such products (Cechin et al., 2023).

All strains analyzed in this study produced the characteristic yellow carotenoid pigment seen in many *Cronobacter* spp., which likely provides protection against desiccation (Johler et al., 2010). All but one (MK_11) synthesized and secreted extracellular polysaccharides in the presence of sucrose, facilitating adhesion and biofilm formation – also on surfaces associated with food production (Iversen et al., 2004; Lehner et al., 2005; Jung et al., 2013; Ogrodzki and Forsythe, 2015). These extracellular polysaccharides also contribute to extended survival in dry food environments and enhance virulence potential (Osaili and Forsythe, 2009). Moreover, all the strains were sensitive to the tested antibiotics, although antibiotic-resistant *Cronobacter* strains (including multidrug-resistant isolates) have previously been identified in food products (Vasconcellos et al., 2018; Li et al., 2019; Arslan and Ertürk, 2021).

One of the *C. sakazakii* strains (MK_10), isolated from dried oregano, was subjected to genomic sequencing and comparative analysis with a clinical isolate (NMI5563_17), obtained from a fatal neonatal infection in Poland. Although these two strains belong to different sequence types (ST31 and ST1, respectively), their genomes exhibited strong synteny and high sequence identity. ST31 strains are detected less frequently in PIF and environmental samples; however, they have also been recovered from severe clinical cases, including fatal infections (Fei et al., 2015; Ogrodzki and Forsythe, 2015). Both MK_10 and NMI5563_17, belonging to different phylogenetic lineages, carry the same set of pathogenesis-associated genes located in their chromosomes and virulence plasmids, although some differences in the gene content were observed. For example, MK_10 lacked some components of the T6SS on both its chromosome and plasmids (variation in the arrangement of these loci has previously been reported in *C. sakazakii*; Wang et al., 2018), while NMI5563_17 lacked several genes associated with the modification of O-antigen, an important pathogenicity factor (Blažková et al., 2015). Notably, MK_10 harbored a putative retron, related to the St85 retron of *Salmonella enterica* (Ahmed and Shimamoto, 2003). Retrons are genetic elements encoding a reverse transcriptase (RT) and a non-coding RNA (ncRNA). The RT uses the ncRNA as a template to generate a chimeric RNA/DNA molecule in which the RNA and DNA components are covalently linked (Millman et al., 2020). The biological role of these enigmatic genetic elements has yet to be fully elucidated. They have been implicated in antiphage resistance (Millman et al., 2020) and have been shown to influence global gene expression, which in *Salmonella Typhimurium* is crucial for anaerobic metabolism required for intestinal colonization (Elfenbein et al., 2015).

The *C. sakazakii* chromosomes contain CRISPR-Cas systems, providing bacteria defense against foreign, invading DNA (Zeng et al., 2017). The MK_10 system is composed of three CRISPR loci and a set of subtype I-E *cas* genes, arranged in a pattern previously reported in *C. sakazakii* species (Zeng et al., 2017). The CRISPR arrays comprise 41 unique spacers, some of which probably target prophages and conjugative plasmids. CRISPR typing is a convenient tool for discriminating *Cronobacter* strains and identifying closely related lineages (Ogrodzki and Forsythe, 2015; Zeng et al., 2020). This procedure identified the closest relatives of MK_10 among *C. sakazakii* strains isolated in the USA.

We also report the presence of functional integrative elements (IE) in *Cronobacter* genomes, carrying adaptive genes that may help their host strains respond to various environmental stresses. These elements, similarly to ICEs and IMEs, contain a site-specific recombination system, although they lack genes required for conjugative transfer. Like many other integrative elements and prophages, the identified IEs integrate into tRNA genes without disrupting their integrity. This permits safe and stable integration into the genome as well as dissemination across a wide range of bacterial species. Both elements exhibit partial similarity to so called “cryptic prophages” initially identified in *E. coli* (Blattner et al., 1997), although they lack genes clearly associated with phage-specific functions. Nevertheless, both elements encode predicted Inovirus Gp2 family proteins (Figures 3B,C), which, although lacking aa similarity, share a domain similar to phage proteins involved in DNA replication (HHpred). This suggests that these elements represent phage satellites (Penadés et al., 2025), although further research is required to confirm this hypothesis.

We designated the MK_10 elements IE*Csak_MK10_1* and IE*Csak_MK10_2* (the proposed nomenclature includes the host strain identifier which provides each element with a unique descriptive name without requiring a centralized nomenclature registry). Structural similarity between these elements is limited to their terminal regions. At one end they carry an integrase gene, while the other contains a type IV TA system of the CbeA/CbeT family, along with several other genes of unknown function. As shown in Figures 3B,C, these genes are arranged in an orientation that may potentially influence the expression of the integrase gene when the element persists in a circular form. The TA activity may promote stabilization of the autonomous form of the element within the bacterial population, as demonstrated for the SXT element (ICE) of *Vibrio cholerae* (Wozniak and Waldor, 2009). Therefore, the co-occurrence of an integrase gene and a CbeA/CbeT-related TA system may serve as a useful marker for the identification and characterization of related IEs in bacterial genomes.

IE*Csak_MK10_1* and IE*Csak_MK10_2* carry different sets of accessory genes. IE*Csak_MK10_1* contains a type III R-M system and several genes involved in DNA metabolism, which, as we hypothesize, may contribute to protection of the bacterial cell against invading foreign DNA. The element also encodes a LeoA/HP0731 family dynamin-like GTPase. A related protein, encoded within an *E. coli* (ETEC) pathogenicity island, has been identified as a virulence factor promoting the release of a heat-labile enterotoxin (LT) from bacterial cells through membrane vesicle-associated secretion (Michie et al., 2014). To our knowledge, related genes have not been previously reported in *Cronobacter* spp.

In contrast, IE*Csak_MK10_2* carries an arsenic resistance gene cluster and encodes two transporters (a SulP family inorganic anion transporter and a phosphoglycerate transporter family protein) as well as a putative universal stress protein (USP). The USP belongs to a widespread family known to enhance bacterial adaptation to various environmental stresses, including fluctuations in temperature, pH, and oxygen availability – conditions commonly encountered during bacterial infections (Chi et al., 2019).

Both IEs also carry the only transposable elements (TEs) identified in the entire MK_10 genome. One of them, located within IE*Csak_MK10_1*, is of particular interest due to its unusual structure. It appears to be a remnant of a Tn*3*-family transposon that has lost the genes required for transposition. However, it retains intact terminal inverted repeats (TIRs), which could potentially be recognized by a compatible transposase encoded by a functional transposon co-occurring in the same cell. This element is flanked on both sides by 5-bp DRs, which is a hallmark of a past transposition event. Such non-autonomous elements are generally not detected in bacterial genomes, as standard TE identification pipelines primarily rely on the presence of transposase genes. The presence of the USP and transporter genes within this non-autonomous transposon highlights its adaptive significance.

The plasmids of the MK_10 strain also exhibit considerable adaptive potential. pCS-MK10_P1 carries genes typical of *C. sakazakii* virulence plasmids, including genes involved in iron acquisition (*eitCBAD* and *iucABCD/iutA*), immune evasion (*cpa*) and chemotaxis (*cmp*). In turn, plasmid pCS-MK10_P2 carries silver efflux and copper resistance determinants, clustered in a conserved and widespread module known as the “copper homeostasis and silver resistance island” (CHASRI). CHASRIs have been shown to confer increased resistance to copper stress under both aerobic and anaerobic conditions, as well as during shifts between the two. Such versatility may explain their prevalence in facultative anaerobes and emerging enteric pathogens (Staehlin et al., 2016). Furthermore, copper has been shown to play a role in macrophage-mediated defense against *Salmonella* infections, suggesting that CHASRIs may provide an additional fitness advantage to pathogenic organisms under host-imposed copper stress (Achard et al., 2012). For this reason, these gene clusters are also referred to as “copper pathogenicity islands” (Hao et al., 2015). It has also been suggested that copper resistance may support the invasion of the central nervous system by *C. sakazakii* during neonatal infections (Kucerova et al., 2010; Singh et al., 2015), although this requires further confirmation.

The concomitant presence of CHASRI and ARS modules in MK_10 suggests that the natural environment of this strain imposed multiple selective pressures involving metal toxicity. CHASRIs are widespread among enteric bacteria. They are found preferentially within plasmids, although they are also commonly observed within transposons of the Tn*7* family as well as in bacterial chromosomes, e.g., in *C. sakazakii* sMOD1-H322 (GenBank: Cp078110; Figure 5B). These islands are often located in close proximity to IS*4* family transposase and HNH nuclease genes, suggesting some role for these enzymes in determining the mobility of the modules (Hao et al., 2015). Notably, in the case of pCS-MK10_P2, the CHASRI is adjacent to an HNH nuclease gene (Figure 5B).

In summary, the MGEs of MK_10 identified and described in this study contain horizontally acquired genes that may enhance the adaptive and potentially pathogenic capabilities of their host strain. The activity of these genes could improve the strain’s tolerance to diverse environmental stressors, including those prevalent in food-processing environments under strong anthropogenic pressure. Better adaptation to such conditions may increase the strain’s survival and proliferation, thereby elevating the risk of food contamination and potential transmission through the food chain. At the same time, the presence of a well-developed CRISPR-Cas system provides defense against foreign DNA, supporting genome integrity and long-term genetic stability, while maintaining a diverse set of pathogenicity-associated genes.

These observations highlight the necessity and importance of conducting in-depth analyses of exogenous DNA introduced into food-borne isolates of opportunistic pathogens, as such events may lead to the emergence of more virulent strains with an enhanced capacity for persistence and dissemination in both natural and anthropogenic environments. These strains may in turn give rise to new evolutionary lineages with the potential for evolutionary success.

## Data Availability

The datasets presented in this study can be found in online repositories. The names of the repository/repositories and accession number(s) can be found at: https://www.ncbi.nlm.nih.gov/genbank/, MW644652-MW644677 and JBSUQN000000000.

## Footnotes

1
http://pubmlst.org/cronobacter/

2
http://www.geospiza.com

3 NCBI; https://ncbi.nlm.nih.gov

4 RDP at the University of Illinois in Urbana, IL; https://rdp.cme.msu.edu

5
https://proksee.ca/
